# Estimated Efficacy of TAK-003 Against Asymptomatic Dengue Infection in Children and Adolescents Participating in the DEN-301 Trial in Asia Pacific and Latin America

**DOI:** 10.1093/infdis/jiaf145

**Published:** 2025-03-18

**Authors:** Tarek El Hindi, Maria Theresa Alera, Lulu Bravo, Edson Duarte Moreira, Reynaldo Dietze, Ana Lucia Oliveira, Veerachai Watanaveeradej, Yuan Zhao, Ivo Sonderegger, Vianney Tricou, Nicolas Folschweiller, Shibadas Biswal

**Affiliations:** Vaccines Business Unit, Takeda Pharmaceuticals International AG, Zurich, Switzerland; Virology Research Unit, Philippines–Armed Forces Research Institute of Medical Sciences, Cebu City; University of the Philippines Manila; Centro de Pesquisa Clínica, Obras Sociais Irmã Dulce and Oswaldo Cruz Foundation, Salvador, Brazil; Escola Superior de Ciências da Santa Casa de Misericórdia de Vitória, Brazil; University of Alabama, Birmingham; Phramongkutklao Hospital, Bangkok, Thailand; Vaccines Business Unit, Takeda Vaccines, Inc., Cambridge, Massachusetts; Vaccines Business Unit, Takeda Pharmaceuticals International AG, Zurich, Switzerland; Vaccines Business Unit, Takeda Pharmaceuticals International AG, Zurich, Switzerland; Vaccines Business Unit, Takeda Pharmaceuticals International AG, Zurich, Switzerland; Vaccines Business Unit, Takeda Vaccines, Inc., Cambridge, Massachusetts

**Keywords:** asymptomatic, dengue, antibodies, TAK-003, vaccine, virus

## Abstract

**Background:**

TAK-003 has been shown to be well tolerated and effective against symptomatic dengue disease and hospitalization, irrespective of baseline serostatus. Most infections are asymptomatic/subclinical. This study assessed whether TAK-003 could protect against asymptomatic/subclinical infections by evaluating increased neutralizing antibody (NAb) titers after natural infection.

**Methods:**

DEN-301 (NCT02747927) is a phase 3 trial among 4- to 16-year-old participants who received 2 doses of TAK-003 or placebo 3 months apart. These exploratory analyses used NAb measured during the trial. As no well-accepted definition for asymptomatic infection exists, 3 algorithms were evaluated: (1) 4-fold increase in NAb, (2) 4-fold increase in NAb and a minimum titer of 40, and (3) 4-fold increase in NAb and a minimum titer of 4-fold lower limit of quantification. Months 4 to 9, months 9 to 15, and months 15 to 27 after first vaccination were analyzed.

**Results:**

NAbs from 3765 participants were analyzed. From months 4 to 9, vaccine efficacy (VE) against asymptomatic infection was 51.1% (95% CI, 30.4%–65.6%), 36.1% (95% CI, 6.7%–56.3%), and 27.3% (95% CI, −8.2% to 51.2%) for algorithms 1, 2, and 3, respectively. VE per algorithms 1, 2, and 3 was 54.8% (95% CI, 28.8%–71.3%), 47.9% (95% CI, 16.8%–67.4%), and 44.3% (95% CI, 9.9%–65.6%) in participants with baseline seropositivity and 44.4% (95% CI, 2.1%–68.4%), 4.6% (95% CI, −85.1% to 50.8%), and −29.3% (95% CI, −172.1% to 38.6%) in those with baseline seronegativity. VE against asymptomatic infection gradually decreased from months 4 to 9 to months 9 to 15 and from months 9 to 15 to months 15 to 27.

**Conclusions:**

The variability in VE algorithms indicates challenges in accurately assessing VE against asymptomatic infections. TAK-003 had a modest impact on asymptomatic dengue infections in the first months postvaccination, mainly in participants with baseline seropositivity.

## Teaser

This study assessed whether TAK-003 could protect against asymptomatic/subclinical dengue infections by evaluating increased neutralizing antibody titers after natural infection. Despite challenges in accurately estimating asymptomatic infections, TAK-003 appeared to have modest efficacy against asymptomatic infections in the first months postvaccination.

The incidence of dengue is increasing worldwide. Dengue is a mosquito-borne disease caused by 1 of 4 antigenically distinct dengue virus (DENV) serotypes: DENV-1, DENV-2, DENV-3, and DENV-4 [1, 2]. Most dengue infections cause no symptoms to mild illness, but some cause severe disease, such as dengue hemorrhagic fever and dengue shock syndrome, and can be fatal if not managed properly [1, 2]. Primary infection with any DENV serotype provides some temporary protection against the other serotypes [2]. However, secondary DENV infection with a different DENV serotype raises the risk to develop severe disease [2].

Approximately 70 years after the first reported outbreak in the Philippines [3], dengue is now endemic in >100 countries [1, 4], and approximately 390 million dengue infections are estimated to occur each year [5]. This number may increase due to urbanization with a lack of urban structure, international mobility, and global warming. Approximately 4 billion individuals are currently at risk for DENV infection globally, and the World Health Organization (WHO) ranked dengue as a grade 3 emergency [6]. Importantly, depending on the study design and methodology, the mean proportion of asymptomatic/subclinical dengue infections among persons infected with dengue varies from 54% to 80% [7]; yet, those with mild or no symptoms will not visit a health care provider, and routine surveillance systems do not detect these cases [8]. Consequently, once a dengue disease case is detected by surveillance systems, the proportion of people who are DENV seropositive may already be high in a population, as reported in dengue-endemic regions [9]. Furthermore, when compared with those infected with symptomatic dengue, asymptomatic individuals who remain active could act as a source of the virus, spreading the infection to mosquitos and subsequently to humans [10, 11]. Additionally, blood donors infected with asymptomatic dengue can transmit DENV directly to humans through transfusion. These donors may travel, which could contribute to the propagation of DENV [12].

Multiple strategies are needed to control the spread of DENV, such as controlling the mosquito populations by removing water containers and focusing on preventing mosquito exposure by using repellents, mosquito nets, traps, and insecticides [13–15]. Notably, the introduction of *Wolbachia* in *Aedes aegypti* mosquito populations, impairing DENV replication in these vectors, was associated with a significant reduction in dengue incidence [16]. However, the sustainability of this strategy needs to be monitored, as *Wolbachia*-resistant DENV variants could emerge over time [17]. To support sustainability, vaccines that can effectively prevent dengue disease could complement vector control and act synergistically to reduce global disease burden [13].

TAK-003 (QDENGA), an available tetravalent recombinant live-attenuated dengue vaccine, was designed to protect persons living in or traveling to dengue-endemic areas against all 4 DENV serotype infections, irrespective of baseline serostatus. The vaccine is based on a DENV-2 backbone that provides a genetic template for the DENV-1, DENV-3, and DENV-4 serotypes [18, 19]. Extensive preclinical and clinical studies led to the pivotal phase 3 DEN-301 trial (TIDES, NCT02747927), which met its primary end point at 1 year after second vaccination with vaccine efficacy (VE) of 80.2% (95% CI, 73.3%–85.3%) against virologically confirmed dengue (VCD). Analyses of key secondary end points revealed that VE against hospitalization was 90.4% (95% CI, 82.6%–94.7%) at 1.5 years postvaccination. Furthermore, VE against VCD disease was comparable among recipients with baseline seronegativity (66.2%; 95% CI, 49.1%–77.5%) and seropositivity (76.1%; 95% CI, 68.5%–81.9) [20, 21]. Latest analyses up to 4.5 years postvaccination showed that cumulative VE was 61.2% (95% CI, 56.0%–65.8%) against VCD (baseline seronegative and seropositive, 53.5% [95% CI, 41.6%–62.9%] and 64.2% [95% CI, 58.4%–69.2], respectively) and 84.1% (95% CI, 77.8%–88.6%) against hospitalized VCD (baseline seronegative and seropositive, 79.3% [95% CI, 63.5%–88.2%] and 85.9% [95% CI, 78.7%–90.7%]) [22]. Overall, the trial demonstrated that TAK-003 was well tolerated and efficacious against dengue disease among individuals aged 4 to 16 years [19–24]. TAK-003 is approved for use in the European Union/European Economic Area and Great Britain, Indonesia, Brazil, Argentina, Colombia, Thailand, Malaysia, Vietnam, Israel, and Switzerland. Regulatory reviews are ongoing in other countries. Additionally, TAK-003 has been prequalified by the WHO for the prevention of dengue disease in individuals from 6 years of age [25], and WHO-SAGE recommended that TAK-003 be considered for public programs in high-transmission areas for those aged 6 to 16 years, without prescreening [26]. In 2024, Brazil initiated a national immunization program with TAK-003 [27].

Although a high VE against symptomatic dengue disease can reduce DENV transmission [28], vaccines that can also prevent asymptomatic/subclinical infections would further decrease transmission. However, in contrast to dengue disease, asymptomatic infections are difficult to detect, so demonstrating VE against asymptomatic dengue infection is challenging [29]. Several studies have shown that increased neutralizing antibody (NAb) titers against DENV during a particular period in the absence of disease is considered a good proxy for asymptomatic infection, and several seroconversion algorithms have been defined [30–36]. Yet, there is currently no universally accepted algorithm to identify asymptomatic infection.

In this analysis, we evaluated data from 3765 participants from the immunogenicity subset of the DEN-301 trial to investigate whether TAK-003 also protected children and adolescents from asymptomatic/subclinical DENV infections by evaluating 3 NAb response seroconversion algorithms over the following periods: months 4 to 9, months 9 to 15, and months 15 to 27.

## METHODS

### Study Design and Participants

**Figure 1.**
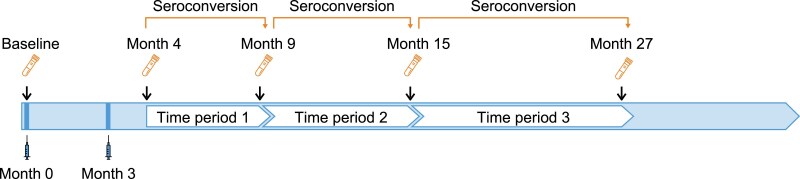
Participants received 2 doses of TAK-003 or placebo at months 0 and 3, and blood samples were collected to assess neutralizing antibody titers from the safety and immunogenicity subset in the DEN301 trial at baseline and months 4, 9, 15, and 27 from the per-protocol set for immunogenicity.

Study details for DEN-301 have been described [20, 23, 24]. This trial was a phase 3 double-blind, placebo-controlled, randomized trial of TAK-003 vs placebo in >20 000 children and adolescents aged 4 to 16 years living in endemic countries (supplementary material). Active surveillance consisted of weekly contact with the participant to identify cases of fever (2 of 3 consecutive days). For exploratory immunologic analyses, this study used NAb titers previously measured from participants administered TAK-003 and placebo from the immunogenicity subset of the trial. NAb titers at baseline and months 4, 9, 15, and 27 were assessed (Figure 1).

### Detection of NAbs

Dengue NAb titers were assessed by a microneutralization assay test for each dengue serotype as described previously [23, 37] (supplementary material). Baseline seropositivity was defined as NAb titers ≥10 against at ≥1 DENV serotype; participants with NAb titers <10 against all 4 serotypes at baseline were considered seronegative. NAb titers below the lower limit of detection (LLOD; <10) were imputed with a value of 5. Actual values were used for values above the lower limit of quantification (LLOQ) and for values between the LLOD and LLOQ (supplementary material).

### Seroconversion Algorithm

A positive seroconversion algorithm for ≥1 DENV serotype was considered asymptomatic DENV infection. Three seroconversion algorithms were evaluated:

Algorithm 1: at least a 4-fold increase in NAb titer from the start of a period to the end.
Algorithm 2: in addition to at least a 4-fold increase in NAb titer, there had to be a minimum threshold of 4 times the LLOD (minimum titer, 40) at the end of the period.
Algorithm 3: in addition to at least a 4-fold increase in NAb titer, there had to be a minimum threshold of 4 times the LLOQ at the end of a period (DENV-1, 4 × 68 = 272; DENV-2, 4 × 87 = 348; DENV-3, 4 × 58 = 232; DENV-4, 4 × 20 = 80).

The 3 algorithms were first evaluated on samples from participants who had had VCD disease during a specific period. After that, data from these participants were excluded from the analysis.

For each seroconversion algorithm described, the relative risk of asymptomatic infection in the TAK-003 group vs the placebo group was calculated as follows: the number of participants with asymptomatic dengue in the TAK-003 group divided by the total number evaluated in the TAK-003 group, over the number with asymptomatic dengue in the placebo group divided by the number evaluated in the placebo group. VE for preventing asymptomatic infection was calculated as 1 minus the odds ratio and presented as the percentage reduction risk of getting asymptomatic infections in the vaccine group as compared with the placebo group. The odds ratio was estimated from a logistic regression model controlling for age and stratified by region (Asia Pacific or Latin America). The efficacy against asymptomatic dengue infection was calculated for the overall group and by baseline serostatus over the following interval periods: months 4 to 9, months 9 to 15, and months 15 to 27.

## RESULTS

### Study Population

The immunogenicity subset of the DEN-301 study consisted of 4000 participants: 1333 in the placebo group and 2667 in the TAK-003 group. The population characteristics were well balanced between the groups (Table 1). Overall, 3765 participants—1247 (93.5%) in the placebo group and 2518 (94.4%) in the TAK-003 group—had no major protocol violations and were included in the analyses. The mean (SD) age was 9.6 (3.35) years. At baseline, 2718 (72.2%) were seropositive for DENV and 1047 (27.8%) were seronegative.

**Table 1.** Population Baseline Characteristics

	TAK-003 Group	Placebo Group	Total
Analysis: per-protocol set for immunogenicity	2518	1247	3765
Age, y, mean (SD)	9.6 (3.36)	9.7 (3.35)	9.6 (3.35)
Age category^a^			
4–5 y			
Overall	316 (12.6)	163 (13.1)	479 (12.7)
Seropositive	184 (10.1)	93 (10.3)	277 (10.2)
Seronegative	132 (18.8)	70 (20.3)	202 (19.3)
6–11 y			
Overall	1386 (55.0)	684 (54.9)	2070 (55.0)
Seropositive	953 (52.5)	481 (53.3)	1434 (52.8)
Seronegative	433 (61.7)	203 (58.8)	636 (60.7)
12–16 y			
Overall	816 (32.4)	400 (32.1)	1216 (32.3)
Seropositive	679 (37.4)	328 (36.4)	1007 (37.0)
Seronegative	137 (19.5)	72 (20.9)	209 (20.0)
Sex			
Male	1275 (50.6)	633 (50.8)	1908 (50.7)
Female	1243 (49.4)	614 (49.2)	1857 (49.3)
Baseline serostatus: overall population			
Seropositive	1816 (72.1)	902 (72.3)	2718 (72.2)
Seronegative	702 (27.9)	345 (27.7)	1047 (27.8)

Data are presented as No. (%) unless specified otherwise.

^a^The proportions of the age groups are based on the total number of participants by baseline serostatus (overall population).

### Evaluation of the Seroconversion Algorithms on Participants Who Had Symptomatic VCD

Seroconversion for all 3 algorithms was detected in 90% (9/10) of participants with VCD from months 4 to 9, 100% (24/24) from months 9 to 15, and 96.9% (31/32) from months 15 to 27 (Table 2). The participant who had symptomatic VCD from months 4 to 9 and did not seroconvert according to the 3 algorithms (a 1.7-fold increase was measured) was in the placebo group, 11 years old, and baseline seropositive. Furthermore, the participant who had symptomatic VCD from months 15 to 27 and did not seroconvert according to the 3 algorithms (a 2.4-fold increase was measured) was in the TAK-003 vaccinated group, 11 years old, and baseline seronegative.

**Table 2.** Confirmation of the 3 Seroconversion Algorithms on Participants Who Had Symptomatic VCD

	TAK-003 (n = 2518)	Placebo (n = 1247)	Total (n = 3765)
Months 4–9			
Participants who had symptomatic VCD	0	10	10
Participants who met all seroconversion algorithms	0	9	9
Months 9–15			
Participants who had symptomatic VCD	13	11	24
Participants who met all seroconversion algorithms	13	11	24
Months 15–27			
Participants who had symptomatic VCD	14	18	32
Participants who met all seroconversion algorithms	13	18	31

Abbreviation: VCD, virologically confirmed dengue.

### Estimated VE of TAK-003 Against Asymptomatic Dengue Infection

**Figure 2.**
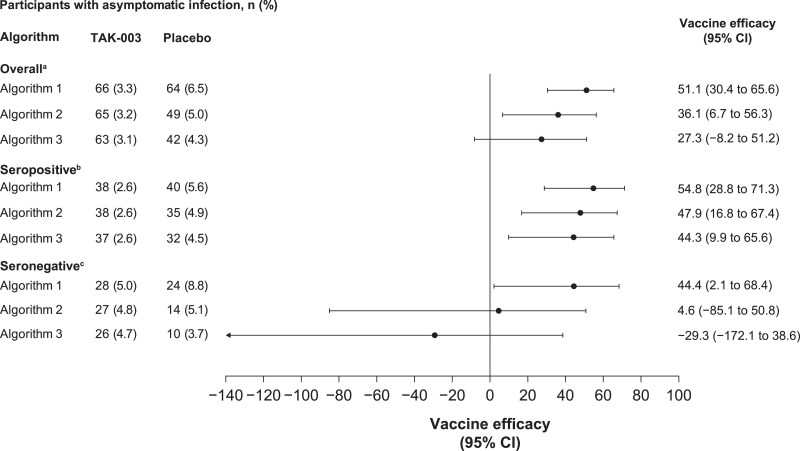
Vaccine efficacy of TAK-003 against asymptomatic dengue infection from months 4 to 9. Algorithm 1, ≥4-fold increase in neutralizing antibodies; algorithm 2, ≥4-fold increase in neutralizing antibodies + ≥40; algorithm 3, ≥4-fold increase in neutralizing antibodies + minimum titer of 4× lower limit of quantification. Percentage of asymptomatic infection is based on the number of participants evaluated in the per-protocol set for immunogenicity. ^a^ Placebo, n = 984; TAK-003, n = 2004. ^b^Placebo, n = 711; TAK-003, n = 1446. ^c^Placebo, n = 273; TAK-003, n = 558.

From months 4 to 9, VE against asymptomatic dengue infection was 51.1% (95% CI, 30.4%–65.6%), 36.1% (95% CI, 6.7%–56.3%), and 27.3% (95% CI, −8.2% to 51.2%) for algorithms 1, 2, and 3, respectively (Figure 2). During this period, VE against asymptomatic dengue infection was higher in participants who were seropositive than seronegative according to all 3 seroconversion algorithms. VE in those who were seropositive according to algorithms 1, 2, and 3 was 54.8% (95% CI, 28.8%–71.3%), 47.9% (95% CI, 16.8%–67.4%), and 44.3% (95% CI, 9.9%–65.6%). In participants who were seronegative, VE according to algorithms 1, 2, and 3 was 44.4% (95% CI, 2.1%–68.4%), 4.6% (95% CI, −85.1% to 50.8%), and −29.3% (95% CI, −172.1% to 38.6%). The first algorithm yielded the highest VE against asymptomatic infections in all groups (Figure 2).

**Figure 3.**
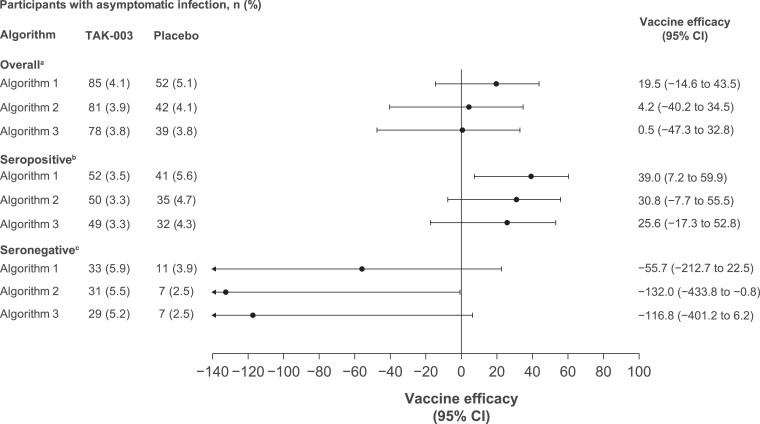
Vaccine efficacy of TAK-003 against asymptomatic dengue infection from months 9 to 15. Algorithm 1, ≥4-fold increase in neutralizing antibodies; algorithm 2, ≥4-fold increase in neutralizing antibodies + ≥40; algorithm 3, 4-fold increase in neutralizing antibodies + minimum titer of 4× lower limit of quantification. Percentage of asymptomatic infection is based on the number of participants evaluated in the per-protocol set for immunogenicity. ^a^Placebo, n = 1022; TAK-003, n = 2055. ^b^Placebo, n = 738; TAK-003, n = 1495. ^c^Placebo, n = 284; TAK-003, n = 560.

From months 9 to 15, VE against asymptomatic dengue infection was reduced as compared with months 4 to 9 and was 19.5% (95% CI, −14.6% to 43.5%), 4.2% (95% CI, −40.2% to 34.5%), and 0.5% (95% CI, −47.3% to 32.8%) for algorithms 1, 2, and 3, respectively (Figure 3). During this period, VE was observed only in participants who were seropositive—at 39.0% (95% CI, 7.2%–59.9%), 30.8% (95% CI, −7.7% to 55.5%), and 25.6% (95% CI, −17.3% to 52.8%) for algorithms 1, 2, and 3—but no longer in those who were seronegative.

**Figure 4.**
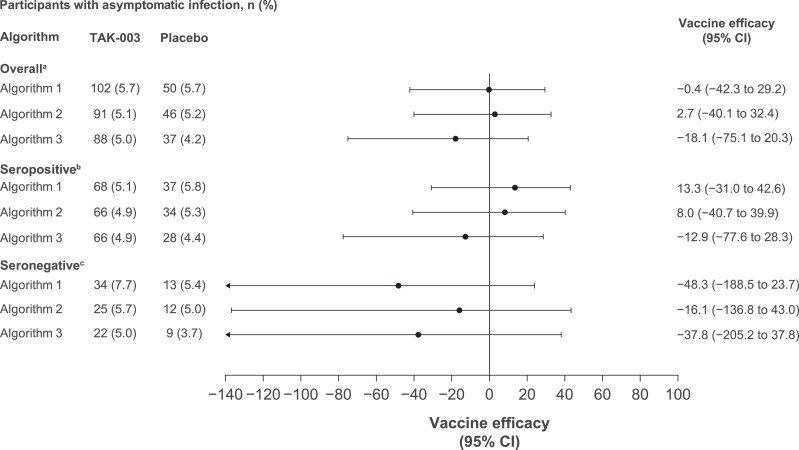
Vaccine efficacy of TAK-003 against asymptomatic dengue infection from months 15 to 27. Algorithm 1, ≥4-fold increase in neutralizing antibodies; algorithm 2, ≥4-fold increase in neutralizing antibodies + ≥40; algorithm 3, ≥4-fold increase in neutralizing antibodies + minimum titer of 4× lower limit of quantification. Percentage of asymptomatic infection is based on the number of participants evaluated in the per-protocol set for immunogenicity. ^a^Placebo, n = 880; TAK-003, n = 1777. ^b^Placebo, n = 638; TAK-003, n = 1337. ^c^Placebo, n = 242; TAK-003, n = 440.

From months 15 to 27, no efficacy was seen as compared with VE at months 9 to 15 in the overall group and was −0.4% (95% CI, −42.3% to 29.2%), 2.7% (95% CI, −40.1% to 32.4%), and −18.1% (95% CI, −75.1% to 20.3%) for algorithms 1, 2, and 3, respectively (Figure 4). Additionally, VE in participants who were seropositive was further reduced to 13.3% (95% CI, −31.0% to 42.6%), 8.0% (95% CI, −40.7% to 39.9%), and −12.9% (95% CI, −77.6% to 28.3%) according to algorithms 1, 2, and 3.

From months 4 to 27—with asymptomatic infections from months 4 to 9, months 9 to 15, and months 15 to 27—the proportion of participants with asymptomatic infections for algorithms 1, 2, and 3 was 85.9% (396/461 infections), 84.7% (360/425 infections), and 83.8% (337/402 infections), respectively. The distribution of asymptomatic infections by country, period, and algorithms is presented in Supplementary Table 1.

### Distribution of NAb Titer Fold Increase

**Figure 5.**
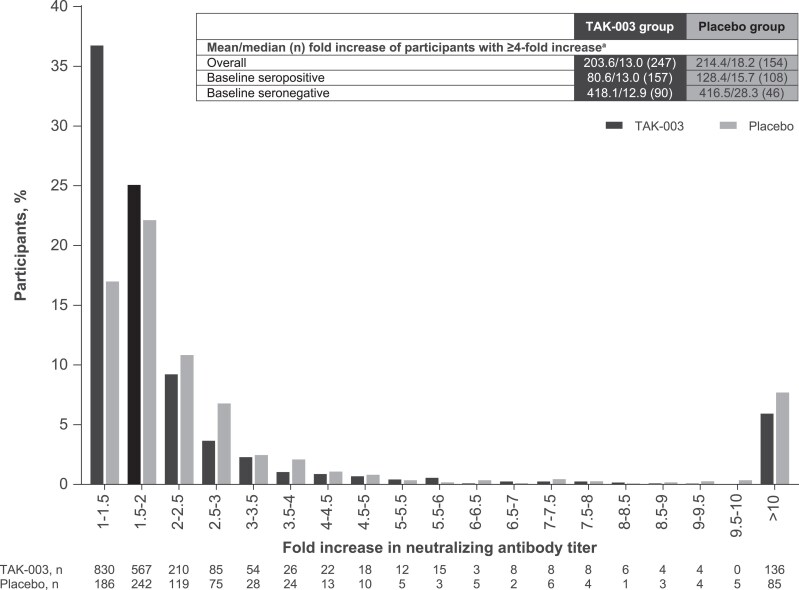
Distribution of neutralizing antibody titer fold increases among participants, excluding virologically confirmed dengue cases, between months 4 and 27 (any increase from months 4 to 9, months 9 to 15, or months 15 to 27 in the per-protocol set for immunogenicity) among TAK-003 vs placebo in baseline participants who were seropositive and seronegative. Participants with increased antibody titers for at least 1 serotype were included in the analysis and are demonstrated in this figure. The algorithms analyzed in this study were based on at least a 4-fold increase in neutralizing antibody titers. The table insert shows the mean and median values of participants who met this threshold. ^a^Virologically confirmed dengue cases were excluded from the analysis, as described in the Methods.

The algorithms evaluated in this study were based on a ≥4-fold increase in NAb titers, as this threshold is sufficiently high to preclude any influence of assay variability (Figure 5). Among participants who had a ≥4-fold increase, excluding those with VCD, the majority had a ≥10-fold increase in antibody titer from months 4 to 27 (placebo, 56.67%; TAK-003, 55.28%; any ≥10-fold increase between months 4 and 9, months 9 and 15, or months 15 and 27). During this period, participants who had a ≥4-fold increase showed higher mean and median fold increases in the placebo group (214.4 and 18.2) as compared with the TAK-003 group (203.6 and 13.0). In the placebo group, participants who were baseline seronegative had higher increases (416.5 and 28.3) than those who were seropositive (128.4 and 15.7). In the TAK-003 group, participants with baseline seronegativity also had a higher mean fold increase (418.1) than those with seropositivity (80.6), but both groups had similar median values (seronegative, 12.9; seropositive, 13.0).

## DISCUSSION

This study evaluated 3 seroconversion algorithms to investigate whether TAK-003 vaccination protected participants from asymptomatic/subclinical DENV infections in the DEN-301 trial. The analysis was based on seroconversion of a ≥4-fold rise, with or without an additional 4-fold LLOD or LLOQ, in NAb from months 4 to 9, months 9 to 15, and months 15 to 27. A minimum of a 4-fold increase in NAb against DENV over a particular time interval without dengue symptoms was also used by previous studies [32, 33]. The results of this study show that although several challenges were observed to precise estimation of asymptomatic infections, TAK-003 appeared to have modest efficacy against asymptomatic DENV infections in the first months after vaccination, with a higher vaccine effect in participants with baseline seropositivity (dengue exposed) as compared with baseline seronegativity (dengue naive). This modest effect after vaccination could still be meaningful in outbreak settings.

From months 4 to 9, VE was observed among the overall group for all 3 algorithms. However, when the population was evaluated by baseline serostatus, VE was observed in participants with baseline seropositivity for all 3 algorithms, while in those with baseline seronegativity, positive VE estimates were limited to algorithms 1 and 2. After 9 months, a gradually decreasing trend in VE was detected in the seropositive populations, and no algorithm was able to demonstrate positive VE estimates in participants with baseline seronegativity. From months 15 to 27, a further reduction in trend was observed in participants with baseline seropositivity, with very low positive estimates demonstrated only in algorithms 1 and 2. Although negative VE estimates were observed in the baseline seronegative population in the periods after 9 months, it is unlikely that TAK-003 recipients with baseline seronegativity have higher chances of asymptomatic infections, as these are primarily driven by exposure and immunity level. One hypothesis for the negative estimates is that asymptomatic infections in TAK-003 recipients with baseline seronegativity may have a quicker and greater immune response as compared with placebo recipients with baseline seronegativity following subsequent DENV exposure due to the vaccine-induced anamnestic memory response. Consequently, titers from TAK-003 recipients who were baseline seronegative were more likely to meet the algorithm for asymptomatic infections.

In the DEN-301 trial, VE was higher and sustained at a higher level against hospitalized VCD cases as compared with all symptomatic VCD cases throughout the study [20–24]. However, VE against asymptomatic infections appears to be modest and short-term as compared with symptomatic infections. Exploratory analysis of the impact of TAK-003 on the clinical profile indicated a favorable effect on the symptoms of dengue and disease severity in participants with VCD [22], suggesting that TAK-003 offers enhanced protection against the clinical manifestations of dengue. A similar pattern of VE was observed in the Dengvaxia VE studies (Sanofi Pasteur) [36, 38]. Moreover, VE studies on SARS-CoV-2 (another unrelated RNA virus) suggest that certain vaccines may mainly confer protection against disease manifestation rather than providing sterilizing immunity. This implies that while vaccination significantly lowers the risk of hospitalization and death, vaccinated persons can still get infected, as real-world data indicate [39, 40]. This pattern of VE suggests that vaccination mainly provides long-term protection against clinical disease symptoms.

A possible explanation for the short-term effect against asymptomatic dengue could be the decline in antibody titers from months 4 to 9 after first vaccination, especially in participants who were seronegative. For longer-term efficacy against asymptomatic infections, a higher persistent magnitude of NAb levels may be required, although the exact mechanism and threshold are unknown. Notably, higher NAb levels were observed in TAK-003 recipients with baseline seropositivity than seronegativity [22]. The higher titer in the seropositive group may explain the greater VE against asymptomatic infections, as the immune systems were already primed by natural dengue exposure, followed by 2 doses of TAK-003. Nevertheless, despite high titers persisting in this population, even after month 15, VE against asymptomatic infection was no longer demonstrated by the seroconversion algorithms.

This study estimated that 84% to 86% of dengue cases are asymptomatic/subclinical, consistent with the range provided by previous studies [7, 8, 41]. The high proportion of asymptomatic infections indicates that the incidence of dengue infection is underreported when using routine surveillance systems by clinical symptoms [8, 41]. However, continued dengue transmission during the postvaccination period in dengue-endemic areas may provide an indirect benefit through natural boosting. In line with this, we previously reported that vaccinated people are less likely to manifest sequential breakthrough dengue over the longer term as compared with placebo (relative risk, 0.19; 95% CI, .07–.54) [42]. We therefore hypothesize that a combination of vaccination and subsequent asymptomatic infections may contribute to persistent efficacy and transition to a postsecondary immunologic state.

This study has limitations. First, it was impossible to virologically confirm infection in asymptomatic participants, as DENV is detectable only for a short period after infection [43], and given the absence of clinical symptoms, this period will pass unnoticed. Second, due to the serologic cross-reactivity of the closely related DENV serotypes, it was not possible to evaluate VE against each serotype, as infection with 1 serotype leads to a rise in titers against other serotypes. Third, although most participants who had symptomatic VCD met all 3 seroconversion algorithms, VE of TAK-003 against asymptomatic dengue infections varied per calculation method, indicating challenges to accurately assessing VE in those with asymptomatic infection. The variation in VE may be due to differences in baseline antibody titers [22] and the magnitude of the response after asymptomatic dengue infection between participants who were baseline seropositive and seronegative, as well as between recipients of TAK-003 and placebo. For example, participants who were seronegative, due to their low baseline titers, were more likely to have a 4-fold rise in antibody titer after infection than those who were seropositive. This is reflected in the higher mean fold increases in participants who were seronegative vs seropositive in the placebo and TAK-003 groups. Within the baseline seronegative group, TAK-003 recipients could have more easily achieved a 4-fold rise than placebo recipients due to the anamnestic response. Furthermore, irrespective of baseline serostatus, TAK-003 recipients had a higher chance of reaching an NAb titer of 40 or a 4-fold LLOQ because of a rise in NAb titer postvaccination. Consequently, the TAK-003 group was more likely to meet the definitions of algorithms 2 and 3, resulting in a lower VE estimate, as compared with algorithm 1. Moreover, the higher threshold of 4-fold LLOQ in algorithm 3 was more likely to be met by participants who were seropositive owing to their elevated titers at baseline [20, 23]. However, it is important to note that the baseline seropositive population is heterogeneous due to the different DENV serotypes and the number of previous dengue infections, which may affect the response to TAK-003 or a natural dengue infection. For instance, although antibody titers are generally higher in participants with baseline seropositivity than seronegativity, for some who were seropositive, achieving an algorithm-specified 4-fold rise in NAb titer may be impossible. Fourth, the blood-sampling schedule in the DEN-301 trial was not planned for VE analyses against asymptomatic infections, and blood samples were not collected at more frequent intervals (eg, every 3 months). More frequent sampling would have increased the precision in NAb kinetics, raising the chance of detecting infections. The longer periods for sampling in the DEN-301 study could have resulted in a lesser chance of meeting the 4-fold increase in antibody titer when infection occurred at the beginning of a period, as antibody titers after infection are expected to wane over time [22].

In conclusion, there are challenges to precisely estimating asymptomatic infections in dengue vaccine trials. Although TAK-003 showed long-term VE against symptomatic dengue, irrespective of baseline serostatus, the vaccine's impact on asymptomatic infections appears to be modest, short-term, and mainly in the baseline seropositive group. Further investigations are warranted to better understand the impact of TAK-003 on dengue transmission.

## Supplementary Material

jiaf145_Supplementary_Data

## Supplementary Data

Supplementary materials are available at *The Journal of Infectious Diseases* online (http://jid.oxfordjournals.org/). Supplementary materials consist of data provided by the author that are published to benefit the reader. The posted materials are not copyedited. The contents of all supplementary data are the sole responsibility of the authors. Questions or messages regarding errors should be addressed to the author.

## Notes

***Acknowledgments.*** We thank the trial participants and their parents/guardians who agreed to take part in the trial, the data-monitoring committee members, the Dengue Case Adjudication Committee members, the Takeda expanded study team, the study team at PPD (part of Thermo Fisher Scientific) who performed monitoring and management of this study, and all the trial staff in each of the countries. Medical writing support was provided by Leonie Huitema, PhD, of Excel Scientific Solutions. We also acknowledge Nicholas Roubinis for analyzing the data during the later stages of manuscript development.

***Author contributions.*** T. E. H.: project administration, visualization, writing–original draft preparation, writing–review and editing. M. T. A.: investigation, project administration, supervision. L. B.: investigation, supervision, writing–review and editing. E. D. M.: conceptualization, investigation, writing–review and editing. R. D.: investigation, writing–review and editing. A. L. O.: investigation. V. W.: investigation, project administration, supervision, writing–review and editing. Y. Z.: formal analysis. I. S.: investigation, project administration, resources, supervision, validation, writing–review and editing. V. T.: conceptualization, investigation, methodology, supervision, writing–review and editing. N. F.: project administration, supervision, writing–original draft preparation, writing–review and editing. S. B.: conceptualization, methodology, project administration, supervision, writing–review and editing.

***Data statement.*** The data sets, including the redacted study protocol, redacted statistical analysis plan, and individual participants' data supporting the results of the completed study, will be made available within 3 months from initial request to researchers who provide a methodologically sound proposal. The data will be provided after its deidentification, in compliance with applicable privacy laws, data protection, and requirements for consent and anonymization. Data requests should follow the process described in the Data Sharing section on https://clinicaltrials.takeda.com and https://vivli.org/ourmember/takeda.

***Financial support.*** This study was supported by Takeda Vaccines, Inc. Medical writing support was funded by Takeda Pharmaceuticals International AG.
