# Supplementary material for: Estimated Efficacy of TAK-003 Against Asymptomatic Dengue Infection in Children and Adolescents Participating in the DEN-301 Trial in Asia Pacific and Latin America
Source: J Infect Dis. 2025 Mar 18;231(6):e1160–9. doi: 10.1093/infdis/jiaf145 (PMC12247829; doi:10.1093/infdis/jiaf145)
Supplement: jiaf145_Supplementary_Data [file jiaf145_supplementary_data.docx]

# Supplementary Material

## Additional Methodology

## Study Details for the DEN-301 Trial

Participants included in the DEN-301 trial were living in 8 dengue-endemic countries across Asia Pacific (Philippines [4 sites], Sri Lanka [4 sites], and Thailand [3 sites]) and Latin America (Brazil [4 sites], Colombia [4 sites], Panama [4 sites], the Dominican Republic [2 sites], and Nicaragua [1 site]). Eligible participants were randomized 2:1 to receive 2 doses of TAK-003 or placebo 3 months apart and have been followed up for 4.5 years. Informed assent or consent forms, and the study protocol and its amendments were reviewed and approved by institutional review boards, independent ethics committees, or health authorities.

During the trial, active surveillance was conducted, and parents/legal guardians of the participants were contacted weekly to remind them to bring their child to the study site if they had febrile illness (defined as ≥ 38°C on two of three consecutive days). This contact was implemented through tailored methods that could have differed between trial sites and included phone calls, text messaging, home visits, and school-based surveillance. If a participant suspected of having dengue returned to the study site, they underwent a thorough clinical assessment by the investigator. Additionally, a blood sample was collected for molecular testing to assess dengue infection, preferably within 5 days of fever onset. A febrile surveillance tracker was used to monitor the incidence of febrile illness and the samples collected, including those that were missed or collected after 5 days of onset of fever.

## Analysis Set

In this study, the Per Protocol Set Immunogenicity (PPSI) subset was used for data analyses. This subset consisted of all randomized participants who received at least 1 dose of TAK-003 or placebo, had a valid blood sample for immunogenicity testing and had no major protocol violations.

## Microneutralization Assay

Dengue NAb titers were assessed by a microneutralization assay test (MNT50, expressed as the reciprocal serum dilution that shows a 50% reduction in plaque count). Individual serially diluted serum samples mixed with DENV were added to a 96-well plate containing Vero cells. After incubation at 2° – 8°C, immunofoci (infectious centers or plaques) were stained using a dengue serotype–specific primary monoclonal antibody and peroxidase-coupled secondary antibody. Foci were quantified in each well. Depending on the serotype-specific primary anti-dengue antibody used to stain immunofoci, the lower limit of quantitation (LLOQ, lower limit with acceptable accuracy and precision) for DENV-1, DENV-2, DENV-3, and DENV-4 was 68, 87, 58 and 20, respectively. MNT_50_ titers were calculated through linear interpolation and represent the reciprocal of the serum dilution that leads to a 50% reduction of plaques compared with the virus control.

**Supplemental Table 1.** Distribution of asymptomatic dengue infections per country and period for all three algorithms (per-protocol set for immunogenicity).

|  | Brazil  N=301 | | Colombia  N=683 | | Dominican  Republic  N=303 | | Nicaragua N=157 | | Panama  N=568 | | Philippines  N=754 | | Sri Lanka  N=369 | | Thailand  N=603 | | **Total**  **N=3765** |
| --- | --- | --- | --- | --- | --- | --- | --- | --- | --- | --- | --- | --- | --- | --- | --- | --- | --- |
| **Months 4**‒**9** | | | | | | | | | | | | | | | | |  |
| Algorithm 1 n, (%) | 12 (4.0) | | 6 (0.9) | | 5 (1.7) | | 5 (3.2) | | 27 (4.8) | | 36 (4.8) | | 15 (4.1) | | 24 (4.0) | | **130** |
| Algorithm 2 n, (%) | 10 (3.3) | | 5 (0.7) | | 5 (1.7) | | 5 (3.2) | | 24 (4.2) | | 32 (4.2) | | 14 (3.8) | | 19 (3.2) | | **114** |
| Algorithm 3 n, (%) | 9 (3.0) | | 3 (0.4) | | 5 (1.7) | | 5 (3.2) | | 21 (3.7) | | 32 (4.2) | | 13 (3.5) | | 17 (2.8) | | **105** |
| **Months 9**‒**15** | | | | | | | | | | | | | | | | |  |
| Algorithm 1 n, (%) | | 4 (1.3) | | 11 (1.6) | | 7 (2.3) | | 5 (3.2) | | 31 (5.5) | | 49 (6.5) | | 10 (2.7) | | 20 (3.3) | **137** |
| Algorithm 2 n, (%) | | 4 (1.3) | | 10 (1.5) | | 7 (2.3) | | 2 (1.3) | | 29 (5.1) | | 46 (6.1) | | 8 (2.2) | | 17 (2.8) | **123** |
| Algorithm 3 n, (%) | | 2 (0.7) | | 9 (1.3) | | 7 (2.3) | | 2 (1.3) | | 28 (4.9) | | 45 (6.0) | | 8 (2.2) | | 16 (2.7) | **117** |
| **Months 15‒27** | | | | | | | | | | | | | | | | |  |
| Algorithm 1 n, (%) | | 10 (3.3) | | 16 (2.3) | | 7 (2.3) | | 1 (0.6) | | 7 (1.2) | | 42 (5.6) | | 23 (6.2) | | 46 (7.6) | **152** |
| Algorithm 2 n, (%) | | 10 (3.3) | | 15 (2.2) | | 7 (2.3) | | 1 (0.6) | | 6 (1.1) | | 37 (4.9) | | 18 (4.8) | | 43 (7.1) | **137** |
| Algorithm 3 n, (%) | | 8 (2.7) | | 14 (2.0) | | 7 (2.3) | | 0 (0) | | 5 (0.9) | | 35 (4.6) | | 17 (4.6) | | 39 (6.5) | **125** |

Algorithm 1, ≥4-fold increase in neutralizing antibodies; Algorithm 2, ≥4-fold increase in neutralizing antibodies + a minimum titer of 40; Algorithm 3, ≥4-fold increase in neutralizing antibodies + minimum titer of 4 × LLOQ. Percentage of asymptomatic infection are based on the number enrolled participants in each country.
